# Pulmonary endarterectomy in antiphospholipid syndrome: a retrospective analysis from the Saudi pulmonary hypertension registry

**DOI:** 10.3389/fmed.2026.1736115

**Published:** 2026-01-14

**Authors:** Noura Alturaif, Fatima K. Alduraibi, Kris Marquez, Fayez Alahmadi, Nadeen Alharbi, Hamdeia Zaytoun, Hanadi Alhamoud, Fatima Alzubi, Mahmoud Hashim, Pekka Hämmäinen, Edward De Vol, Abdullah M. Aldalaan

**Affiliations:** 1 Pulmonary Hypertension, Department of Lung Health, Organ Transplant Centre, King Faisal Specialist Hospital and Research Centre, Riyadh, Saudi Arabia; 2 Division of Rheumatology, Department of Medicine, King Faisal Specialist Hospital and Research Centre, Riyadh, Saudi Arabia; 3 Division of Rheumatology, Department of Medicine, Beth Israel Deaconess Medical Centre, Harvard Medical School, Boston, MA, United States; 4 Analytics Data Centre, Organ Transplant Centre, King Faisal Specialist Hospital and Research Centre, Riyadh, Saudi Arabia; 5 Thoracic Surgery, Department of Lung Health, Organ Transplant Centre, King Faisal Specialist Hospital and Research Centre, Riyadh, Saudi Arabia; 6 Cardiothoracic Transplant Unit, Royal Papworth Hospital, Cambridge, United Kingdom; 7 Vascular Service, Department of Surgery, Terveystalo Hospital, Helsinki, Finland; 8 Department of Biostatistics, Epidemiology and Scientific Computing, King Faisal Specialist Hospital and Research Centre, Riyadh, Saudi Arabia

**Keywords:** autoimmunity, chronic thromboembolic pulmonary hypertension, outcomes, persistent pulmonary hypertension, surgery

## Abstract

**Background:**

Antiphospholipid syndrome (APS) is associated with venous thromboembolism, which can lead to chronic thromboembolic pulmonary hypertension (CTEPH). Despite treatment with pulmonary endarterectomy (PEA), some patients continue to experience pulmonary hypertension (PH), which is potentially caused by APS-related distal vasculopathy. The aim of this study was to assess persistent PH after surgery and to investigate post PEA outcomes, including immediate postoperative complications, hospitalizations within 1 year of surgery, and mortality.

**Methods:**

We performed a retrospective analysis of adult patients with CTEPH who underwent PEA. We included patients with and without APS. Data were obtained from the Saudi Pulmonary Hypertension Registry (2011–2022). We assessed immediate postoperative complications, rehospitalizations within 1 year, and mortality. Additionally, we evaluated patients with right heart catheterization 1 year before and after PEA for persistent PH.

**Results:**

The study included 37 patients with APS-CTEPH and 41 patients without APS. Persistent PH was observed in 43% of patients, with no significant difference between the groups. Notably, patients in the APS-CTEPH group who received pulmonary vasodilator therapy before PEA had a reduced risk of developing persistent PH. Postoperative complications were significantly higher in the APS-CTEPH group (*p* = 0.002). Despite these risks, the 5-year survival rate was 98.3%, with no significant difference between the groups.

**Conclusion:**

APS is associated with a higher incidence of immediate postoperative complications and rehospitalizations within the 1^st^ year after surgery. However, APS does not appear to increase the risk of persistent PH or affect long-term mortality in this cohort.

## Graphical abstract

**Figure:**
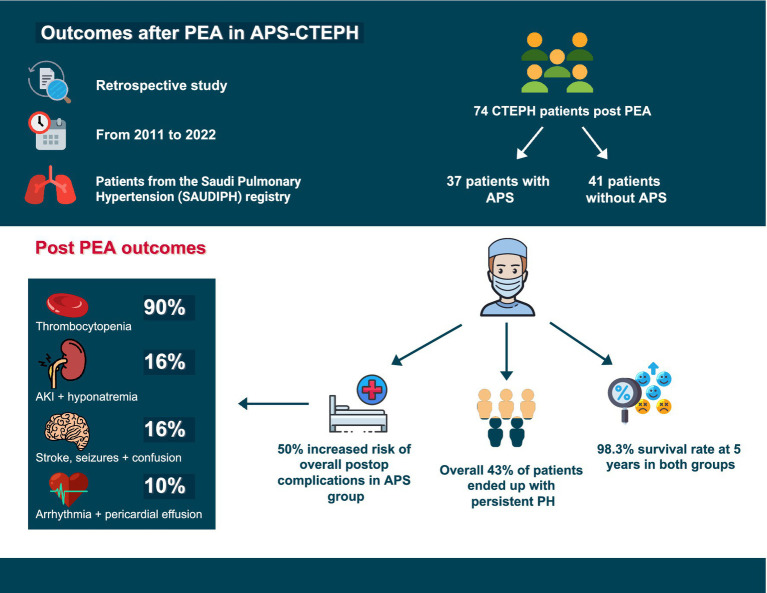

## Introduction

Antiphospholipid syndrome (APS) is a complex autoimmune disorder characterized by the persistent presence of antiphospholipid antibodies and a history of arterial or venous thrombotic events or specific pregnancy-related complications (1). APS often impacts the lungs in various ways, with pulmonary embolism (PE) being the most prevalent pulmonary manifestation (2). Throughout the disease course, 14.1% of APS patients experience PE (2, 3).

Pulmonary hypertension (PH) has emerged as the second most common pulmonary complication in patients with APS (4). The prevalence of PH in APS patients is 3.5% in those with primary APS and 1.8% in those with secondary APS (5). Chronic thromboembolic pulmonary hypertension (CTEPH) is notably prevalent among patients with APS because of their increased susceptibility to PE. Following PE, the incidence of developing CTEPH is estimated to be 3% (6, 7). The follow-up after acute PE trial reported a 2-year cumulative incidence of 2.3% (8). Additionally, APS is identified in 20% of CTEPH patients (9).

While various medical and interventional therapies are available, pulmonary endarterectomy (PEA) is the only curative treatment. PEA is the most effective treatment for patients with CTEPH who have operable disease (10). Patients who undergo PEA have significantly higher survival rates than those who do not undergo PEA (11, 12). Despite prior studies confirming the safety of PEA in patients with APS and describing immediate postoperative complications, there is a notable lack of data on long-term outcomes, specifically for the persistence or recurrence of PH after treatment with PEA (13–15).

In light of this, we hypothesized that patients diagnosed with APS and CTEPH (APS-CTEPH) may display distinct clinical characteristics and outcomes compared with patients diagnosed with CTEPH without APS (non-APS CTEPH) due to the underlying autoimmune conditions and chronic inflammation. This unique profile might result in more severe distal vasculopathy and vascular remodeling as well as World Health Organization Group I pulmonary arterial hypertension features, which could lead to a greater likelihood of persistent or recurrent PH following PEA, irrespective of the surgical technique employed. This scenario could result in a poorer long-term prognosis and increased mortality rate for patients with APS-CTEPH than for those with non-APS CTEPH.

Our study compared the baseline characteristics of the APS-CTEPH and non-APS CTEPH groups, investigated the short-term and long-term outcomes, specifically the risk of persistent disease, following PEA in each group, and identified predictors of adverse outcomes, particularly within the APS-CTEPH cohort.

## Methods

### Study design and population

We conducted a retrospective analysis of adult patients diagnosed with APS-CTEPH and non-APS CTEPH at our institution. Patients who had undergone PEA from 2011 to 2022 were identified from the Saudi Pulmonary Hypertension registry. The electronic health records were reviewed for potential inclusion in the final study population. The study was conducted in compliance with the Helsinki Declaration and was approved by the Institutional Review Board (RAC # 2231137).

We included adults over 18 years with APS-CTEPH or non-APS CTEPH who underwent PEA. APS was diagnosed per the American College of Rheumatology/European League Against Rheumatism criteria, and patients were seen at least once by a board-certified rheumatologist (16). The diagnosis of APS relies on clinical suspicion such as unexplained arterial or venous thrombosis or specific pregnancy morbidity followed by confirmatory laboratory testing. All patients must have at least one positive APS test (lupus anticoagulant, anticardiolipin antibodies [IgG and/or IgM, medium–high titer], or anti–*β*₂-glycoprotein I antibodies [IgG and/or IgM, medium–high titer]), and positivity must be persistent on two occasions at least 12 weeks apart (17). APS is confirmed when one clinical criterion and one persistent laboratory criterion are both present (17). CTEPH diagnosis required chronic clots on imaging (ventilation/perfusion scan and computed tomography chest angiogram) that was confirmed by a chest radiologist, and a PH diagnosis via right heart catheterization (RHC) per the 2022 ESC/ERS guidelines (12). Patients were excluded if they were in World Health Organization groups other than Group IV or had thrombophilia disorders except for APS. A total of 86 patients with CTEPH post-PEA were identified. Four were lost to follow-up, three had other hyperproliferative diseases (Factor V Leiden, protein C, and S deficiency), and one had combined CTEPH and congenital heart disease. The final number of patients included was 78.

### Data collection

The following data were extracted from the electronic health records: Demographics; comorbidities; antiphospholipid status (primary vs. secondary); six-minute walk distance; computed tomography angiogram findings; echocardiography metrics; and hemodynamic parameters based on RHC. The clinical data collected included persistent PH, which was defined in our study as a mean pulmonary arterial pressure (mPAP) greater than 30 mmHg and pulmonary vascular resistance (PVR) over 2 Wood units. Given the lack of clearly established cutoffs for defining persistent pulmonary hypertension or guiding treatment initiation following pulmonary endarterectomy, we adopted a mPAP threshold of 30 mmHg, consistent with clinical practice and the level at which most pulmonary hypertension experts would consider initiating pulmonary vasodilator therapy (18, 19). These parameters were measured during the first postoperative RHC within the 1st year after surgery. Notably, prior studies, including a meta-analysis published in 2018, have highlighted the lack of a consistent or universally accepted definition of persistent disease (20).

Postoperative complications were categorized into hematological (thrombocytopenia), metabolic (hyponatremia and acute kidney injury), neurological (seizures, confusion, and strokes), and cardiac (arrhythmia and pericardial effusion/tamponade). This classification was based on existing literature that systematically details the spectrum of complications observed in patients with APS undergoing cardiac surgeries (21–24). Hematological and metabolic changes were considered surgery-related if they were observed within 5 days post-surgery. Thrombocytopenia was defined as a platelet count of less than 150 × 10 (9)/L, while severe thrombocytopenia was defined as a platelet count of less than 50 × 10 (9)/L. Hyponatremia was defined as a sodium level of less than 135 mEq/L. Complications were recorded as either absent or present, with the latter indicating the occurrence of one or more complications. We collected the number of hospitalizations/events after discharge from PEA admission during the 1st year following discharge. Additionally, we collected data on changes in hemodynamics before and after surgery via RHC data collected within 1 year before and after surgery.

### Statistical analysis

We used R software for statistical analysis. Descriptive data were presented as means and standard deviations for continuous variables and as frequencies and percentages for categorical variables. Statistical significance was set at a *p* value of < 0.05. The Mantel–Haenszel method estimated risk or odds ratios. Kaplan–Meier curves were used for survival analysis from the surgery date until death.

We compared immediate postoperative complications, hospitalizations within 1 year after surgery, persistent PH, and mortality between the APS-CTEPH and non-APS CTEPH groups. Outcomes were adjusted for comorbidities, thromboembolic events, and pre-surgery pulmonary vasodilator therapy. The risk of hospitalization within 1-year post-discharge from PEA admission and the survival rate after surgery were analyzed. To more accurately evaluate persistent PH and hemodynamic changes before and after surgery, we included only the hemodynamic data from patients who underwent RHC within 1 year before and after surgery. This criterion was met by 40 out of 74 patients, and the others were excluded from this part of the analysis.

## Results

### Patient characteristics

Our study included 37 patients with APS-CTEPH and 41 patients with non-APS CTEPH who underwent PEA between 2011 and 2022 (Table 1). Our patients were predominantly female (73%). The mean age was 34.5 (± 10.9) years in the APS-CTEPH group and 33 (± 10.7) years in the non-APS CTEPH group. Most patients were classified as New York Heart Association functional class II-III. More than 50% of patients in both groups were receiving pulmonary vasodilator therapy before surgery. The history of venous thromboembolism episodes is detailed in Table 1. Among patients with APS-CTEPH, 86% demonstrated double or triple antiphospholipid antibody positivity, equally distributed between double (43%) and triple (43%) positivity. Interestingly, systemic lupus erythematosus was the only connective tissue disease associated with secondary APS in our cohort, consistent with its well-recognized association as the most common CTD linked to APS (1, 25). Approximately 65% of patients with APS-CTEPH were treated with immunosuppressive and/or immune-modulating therapy. Hydroxychloroquine was the most common type of therapy (Supplementary Tables 1, 2). The patients underwent surgery at various locations including 78% of patients at our center, 19% in the United States, and 3% in Europe.

**Table 1:** Baseline characteristics of patients diagnosed with chronic thromboembolic pulmonary hypertension with and without antiphospholipid syndrome before pulmonary endarterectomy.

Baseline characteristics	APS-CTEPH, *n* (% among *n* = 37)	Non-APS CTEPH, *n* (% among *n* = 41)
Age at CTEPH diagnosis	34.5 ± 9.5	33 ± 10.61
Sex		
Female	26 (70.0)	31 (75.6)
Male	11 (30.0)	10 (24.4)
Comorbidities		
DM	3 (8.1)	3 (7.3)
HTN	2 (5.4)	5 (12.2)
Heart failure	4 (10.8)	1 (2.4)
CKD	4 (10.8)	3 (7.3)
Seizures	3 (8.1)	1 (2.4)
CTD	10 (27.0)	0 (0)
NYHA FC at CTEPH diagnosis		
FC I	0 (0)	1 (2.4)
FC II	11 (30.0)	13 (31.7)
FC III	20 (54.0)	21 (51.2)
FC IV	6 (16.0)	6 (14.6)
History of DVT only		
Single	0 (0)	0 (0)
Multiple	2 (5.4)	2 (6.0)
History of PE only		
Single	9 (24.3)	19 (54.0)
Multiple	2 (5.4)	7 (20.0)
History of DVT and PE		
Single DVT and PE	8 (21.6)	4 (11.0)
Multiple DVTs and single PE	4 (10.8)	1 (3.0)
Multiple PEs with ≥ 1 DVT	10 (27.0)	2 (6.0)
ProBNP at PEA in pg./ml	1,002 ± 1,014	810 ± 901
Most recent 6MWT before PEA	202 ± 151	219 ± 118
Hemodynamics at CTEPH diagnosis		
RA in mmHg	14.47 ± 6.50	12.38 ± 5.95
mPAP in mmHg	51.00 ± 20.00	52.72 ± 11.28
PAWP in mmHg	13.77 ± 4.41	13.68 ± 6.13
PVR in Wood Units	12.05 ± 7.00	12.42 ± 7.52
CO in L/min/CI	3.29 ± 0.83	3.74 ± 1.26
CI in L/min/m^2^	1.87 ± 0.35	1.97 ± 0.60
SvO2%	56.00 ± 10.00	57.58 ± 9.64
Most recent echocardiography before PEA		
RV size		
Normal	4 (10.8)	5 (12.5)
Mild dilation	6 (16.2)	3 (7.5)
Moderate dilation	10 (27.0)	8 (20.0)
Severe dilation	17 (46.0)	24 (60.0)
RV function		
Normal	4 (11.0)	9 (22.5)
Mild dysfunction	11 (30.0)	5 (12.5)
Moderate dysfunction	10 (27.0)	10 (25.0)
Severe dysfunction	12 (32.0)	16 (40.0)
CT chest findings before PEA		
Acute on chronic PE	3 (8.3)	4 (10.5)
Mosaic attenuation	28 (77.8)	37 (97.4)
Pulmonary infarctions	7 (19.4)	7 (18.4)
Collaterals	9 (25.0)	0 (0)
Pulmonary vasodilator therapies		
On therapy	20 (54.0)	26 (63.5)
Monotherapy	9 (24.0)	11 (27.0)
Combination	11 (30.0)	15 (36.5)
Type of pulmonary vasodilator therapy		
Sildenafil	14 (70.0)	16 (61.5)
Bosentan	6 (30.0)	9 (34.6)
Macitentan	5 (25.0)	7 (27.0)
Riociguat	3 (15.0)	6 (23.0)
Iloprost	5 (25.0)	2 (7.6)
Type of AC		
DOAC	4 (11.0)	23 (56.1)
Warfarin	30 (81.0)	16 (39.0)
LMWH	3 (8.0)	2 (4.9)
History of noncompliance with AC	2 (5.4)	1 (2.4)

6MWT, 6-min walk test; AC, Anticoagulation; APS, Antiphospholipid syndrome; CI, Cardiac index; CKD, Chronic kidney disease seizures; CO, Cardiac output; CT, Computed tomography; CTD, Connective tissue disease; CTEPH, Chronic thromboembolic pulmonary hypertension; DM, Diabetes mellitus; DOAC, Direct oral anticoagulants; DVT, Deep vein thrombosis; FC, Functional class; HTN, Hypertension heart failure; LMWH, Low molecular weight heparin; mPAP, Mean pulmonary artery pressure; NYHA, New York Heart Association; PAWP, Pulmonary artery wedge pressure; PE, Pulmonary embolism; PEA, Pulmonary endarterectomy; ProBNP, N-terminal pro b-type natriuretic peptide; PVR, Pulmonary vascular resistance; RA, Right atrium; RV, Right ventricle; SvO2, Mixed venous oxygen saturation.

**Figure 1:**
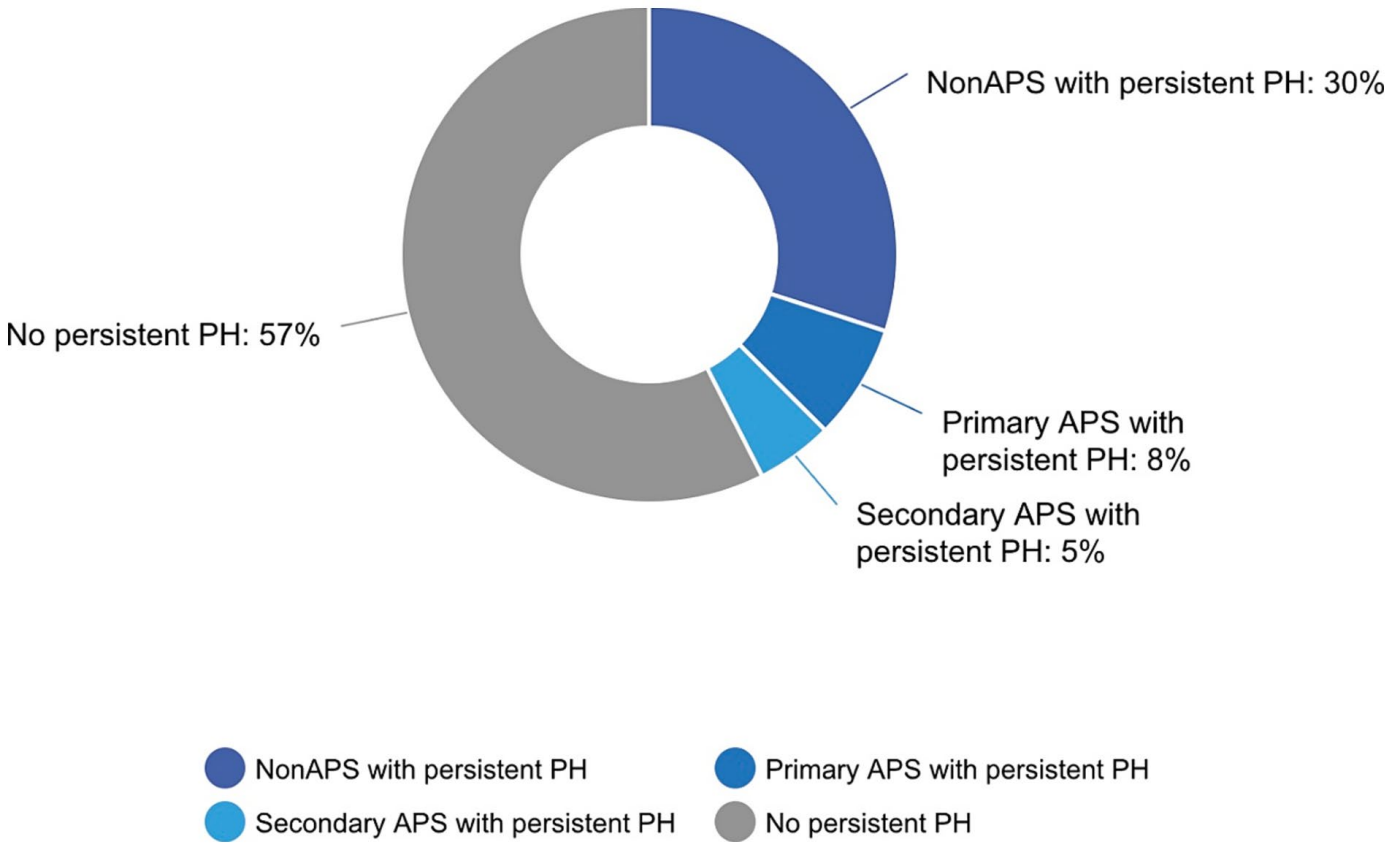
Percentage of patients with persistent pulmonary hypertension (PH) according to antiphospholipid syndrome (APS) status.

To evaluate persistent PH after PEA, we included patients who underwent RHC within the 1st year, with a focus on persistent rather than recurrent PH. Approximately 43% of patients exhibited persistent disease, with no significant difference between the APS-CTEPH and non-APS CTEPH groups (*p* = 0.5) (Figure 1). Notably, the risk ratio for patients with APS-CTEPH receiving pulmonary vasodilator therapy before PEA was 0.52 [95% confidence interval (CI): 0.28–0.97] and was 2.65 (95%CI: 0.63–11.19) for those not receiving therapy. These values were significantly different from each other (*p* = 0.04). Approximately 32% of patients in both groups were on combination therapy after surgery.

### Postoperative complications, hospitalizations, and mortality

**Figure 2:**
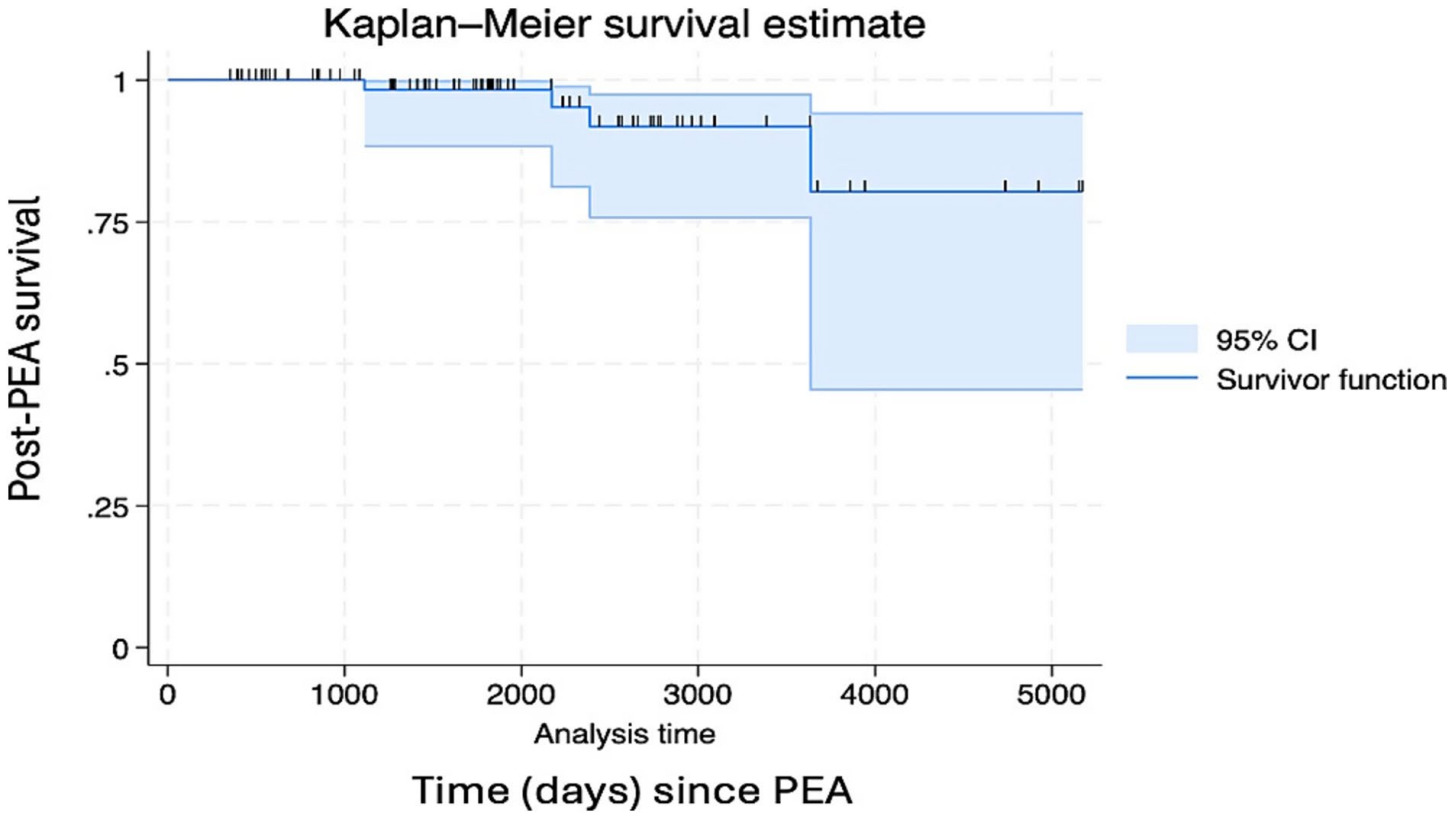
Post-surgery survival of the overall chronic thromboembolic pulmonary hypertension (CTEPH) group.

**Figure 3:**
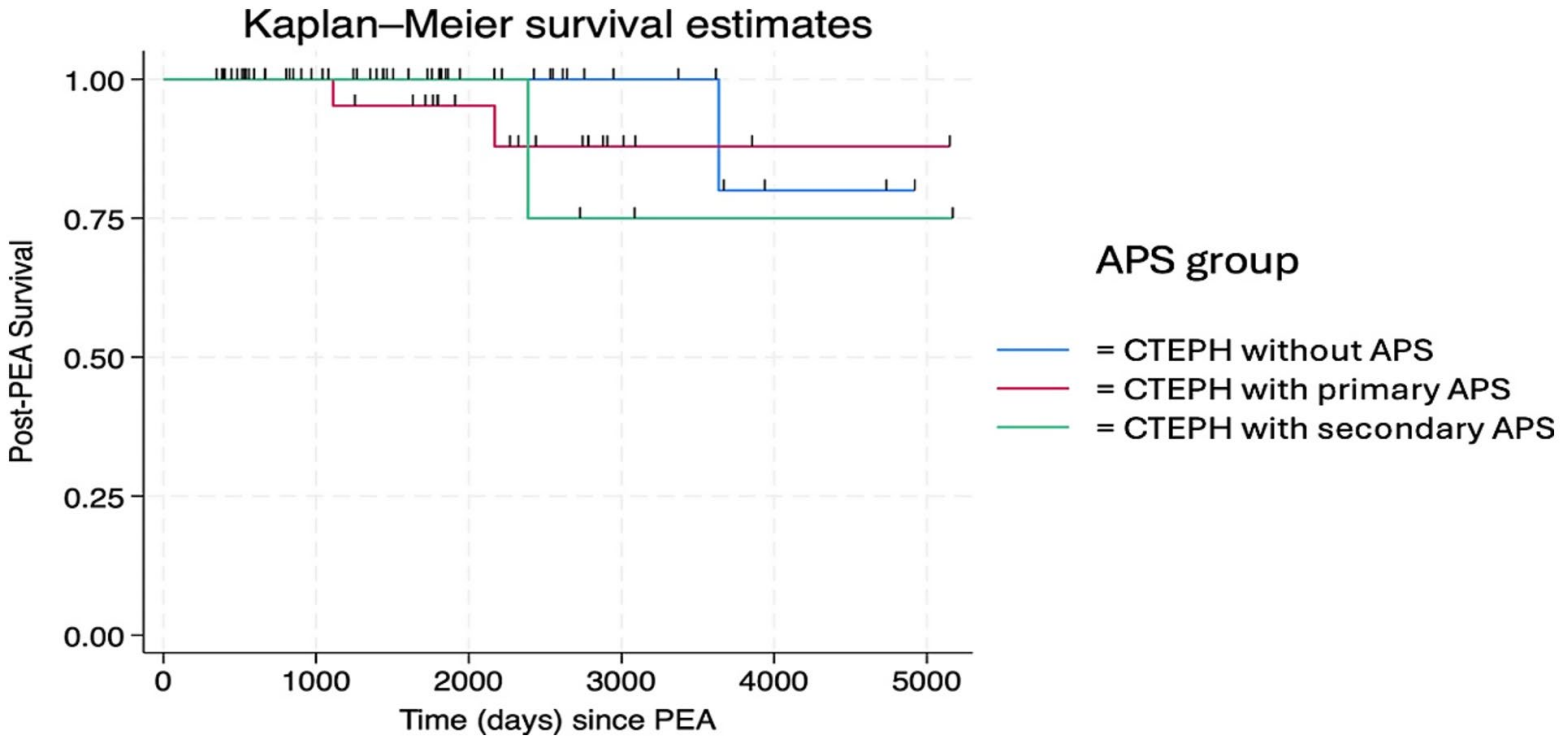
Post-surgery survival of chronic thromboembolic pulmonary hypertension (CTEPH) subgroups according to antiphospholipid syndrome (APS) status: non-, primary, and secondary.

Postoperative hematological, metabolic, neurological, and cardiac complications were analyzed. Patients with non-APS CTEPH had a significantly lower risk of post-PEA complications (60.0%) than patients with APS-CTEPH (93.6%), which corresponded to a risk ratio of 1.56 (95%CI: 1.15–2.12; *p* = 0.002). Thrombocytopenia was common in both groups after surgery (90% in the APS-CTEPH group vs. 45% in the non-APS CTEPH group) but was more severe in the APS-CTEPH group. Renal and neurological complications occurred in 16% of patients with APS-CTEPH, and cardiac complications occurred in 10% of patients with APS-CTEPH. Pulmonary vasodilator therapy before PEA did not significantly impact the occurrence of complications (*p* = 0.11). Additionally, hospitalization rates within the 1st year after surgery were higher in the APS-CTEPH group (*p* = 0.016). Mortality post-PEA was recorded at 3.0 years, 5.9 years, 6.5 years, and 10.0 years, with a 5-year survival rate of 98.3% and a lower confidence limit for true survival of 88.4%, with no statistically significant differences (*p* = 0.26 between the APS-CTEPH and non-APS CTEPH groups) (Figures 2, 3).

### First hemodynamic evaluation after PEA

Both groups exhibited statistically significant improvements in hemodynamics following surgery (*p* < 0.0001). Specifically, there was a 29% decrease in mPAP, a 54% decrease in PVR, a 19% increase in the cardiac index, and an 18% increase in mixed venous saturation (SvO2). No significant differences were observed between the two groups except for the change in SvO2 in the APS-CTEPH group (*p* = 0.02) (Table 2 and Supplementary Table 3).

**Table 2:** Characteristics of patients diagnosed with chronic thromboembolic pulmonary hypertension with and without antiphospholipid syndrome after pulmonary endarterectomy.

Characteristics of post-PEA	APS-CTEPH, *n* (% among *n* = 37)		Non-APS CTEPH, *n* (% among *n* = 41)
	Primary (*n* = 27)	Secondary (*n* = 10)	
Age in years at PEA surgery	36.7 ± 10.0	35.0 ± 9.0	37.9 ± 11.0
Days from diagnosis to PEA surgery	257 ± 593	652 ± 1,147	349 ± 423
Site of PEA surgery			
KSA	24	7	30
USA	2	2	11
Europe	1	1	0
Hemodynamics at first follow-up RHC			
RA in mmHg	9.0 ± 4.0	11.0 ± 4.0	10.0 ± 4.4
mPAP in mmHg	32.0 ± 10.0	27.0 ± 6.5	34.0 ± 14.0
PAWP in mmHg	12.0 ± 2.6	14.0 ± 3.2	14.0 ± 5.0
PVR in Wood units	5.7 ± 3.6	3.4 ± 2.0	4.0 ± 1.1
CO in L/min	3.9 ± 1.1	4.0 ± 1.1	4.0 ± 1.1
CI in L/min/m^2^	2.0 ± 0.4	2.2 ± 0.5	2.2 ± 0.5
SvO_2_%	64.0 ± 7.0	65.0 ± 6.0	65.5 ± 7.0
Echocardiography at follow-up			
RV size			
Normal	9 (33)	5 (50)	16 (39)
Mild dilation	11 (41)	3 (30)	13 (32)
Moderate dilation	5 (19)	2 (20)	7 (17)
Severe dilation	2 (7)	0 (0)	5 (12)
RV function			
Normal	8 (30)	4 (40)	11 (27)
Mild dysfunction	11 (41)	2 (20)	15 (37)
Moderate dysfunction	6 (22)	4 (40)	10 (24)
Severe dysfunction	2 (7)	0 (0)	5 (12)
Pulmonary vasodilator therapies			
Monotherapy	9 (33)	3 (30)	9 (22)
Combination	6 (22)	1 (10)	13 (32)
Type of pulmonary vasodilator therapy			
Riociguat	9 (60)	0 (0)	17 (77)
Macitentan	9 (60)	4 (100)	16 (73)
Sildenafil	3 (20)	1 (25)	2 (9)
Selexipag	0 (0)	0 (0)	2 (9)
Iloprost	0 (0)	0 (0)	1 (5)
SC Treprostinil	1 (10)	0 (0)	0 (0)
Type of AC			
DOAC	2	2	24
Warfarin	23	6	17
Enoxaparin	2	2	0

AC, Anticoagulant; APS, Antiphospholipid syndrome; CO/CI, Cardiac output/cardiac index; CTEPH, Chronic thromboembolic pulmonary hypertension; DOAC, Direct oral anticoagulant; KSA, Kingdom of Saudi Arabia; mPAP, Mean pulmonary artery pressure; PAWP, Pulmonary artery wedge pressure; PEA, Pulmonary endarterectomy; PVR, Pulmonary vascular resistance; RA, Right atrium; RHC, Right heart catheterization; RV, Right ventricle; SvO2, Mixed venous oxygen saturation; USA, United States of America.

## Discussion

In this study, we assessed the immediate and long-term outcomes of patients diagnosed with CTEPH both with and without APS after treatment with PEA. The patients in the APS-CTEPH group in our study were young, which is consistent with the literature (13). However, the patients in the non-APS CTEPH group were significantly younger than those previously reported in Western countries (26). Our findings showed that treatment with PEA resulted in significant hemodynamic improvements in both groups. However, the APS-CTEPH group demonstrated a statistically significant improvement in SvO2 compared with the non-APS CTEPH group (*p* = 0.02), suggesting a greater impact on cardiac function and tissue oxygenation in patients with APS.

Patients in the APS-CTEPH group faced a significantly greater risk of immediate postoperative complications than patients in the non-APS CTEPH group. The risk ratio was 1.56 (95%CI: 1.15–2.12; *p* = 0.002). This represents an approximately 50% increased risk for the patients in the APS-CTEPH group. Postoperative thrombocytopenia was significantly more prevalent in the APS-CTEPH group, affecting 90% of patients. We observed that 22.5% of patients experienced a severe case where platelet counts fell below 50 × 10 (9)/L, and interventions such as corticosteroids and intravenous immunoglobulin were required. This complication, observed exclusively in the APS-CTEPH group, presented considerable challenges due to the need for continuous anticoagulation and the heightened risk of bleeding. Additionally, more serious complications in the APS-CTEPH group included neurological issues such as stroke or confusion. Hyponatremia was common but not severe, as indicated by a nadir postoperative sodium level of 130 mEq/L. Our findings aligned with the current literature (14, 15, 27). However, we found that an increased risk of postoperative complications led to a higher rate of hospitalization within the 1st year after surgery. In our cohort, 9% of patients required hospitalization within the 1^st^ year after undergoing PEA. Hospitalization causes included APS flare, cerebellar hematoma, pericardial effusion/tamponade, and sepsis.

Previous studies have linked autoimmunity in APS with distal vasculopathy. It has been observed that pulmonary arterial hypertension has been thought to initially be CTEPH but was later found to be associated with primary APS (28–30). We hypothesized that patients in the APS-CTEPH group would have an increased risk of developing persistent PH after surgery. In our cohort of 37 patients with APS-CTEPH, nearly half had triple antiphospholipid antibody positivity, indicating a highly thrombogenic group (17). Although 43% of patients had persistent PH at their first postoperative follow-up RHC within the 1st year after PEA, there was no significant difference between the APS-CTEPH and non-APS CTEPH groups. Data from U. S. and European CTEPH registries show that persistent PH after PEA occurs in a proportion of patients, with reported prevalence ranging from approximately 17 to 51%, depending on the definition and timing of reassessment (18, 31). In the International CTEPH Registry, Hoeper et al. reported persistent PH in 16.7% of patients, defined as mPAP ≥25 mm Hg at the end of intensive care (31). Rates of persistent PH increase with later evaluation and stricter hemodynamic criteria, reaching 31% at 1 year post-PEA in the European cohort reported by Skoro-Sajer et al. (33) and 51% at 3–6 months in the UK National CTEPH Cohort (18). Similarly, Freed et al. reported elevated mPAP (≥30 mm Hg) at 3 months post-PEA in 31% of patients (32). Persistent PH was observed in 43% of our patients, which lies toward the higher end of rates reported in international registries. Potential reasons include a distinct patient population characterized by a high prevalence of APS, prolonged disease duration prior to referral, and a high operability rate, with approximately 75% of CTEPH patients undergoing PEA during a period when PEA was the primary therapeutic intervention at our center. Surgical specimen classification is not available for our patients, precluding further characterization of disease distribution or assessment of whether a substantial proportion had predominantly distal disease contributing to persistent pulmonary hypertension. This is clinically relevant given prior data from a Canadian cohort, which reported residual pulmonary hypertension in up to 38% of patients with CTEPH after PEA, particularly among those with more distal disease involvement (type 3 disease, originating at the segmental pulmonary artery branches) (33). Postoperatively, patients with persistent PH were treated with pulmonary vasodilator therapy, as balloon pulmonary angioplasty was not available during the study period and was only established in 2025.

Interestingly, patients in the APS-CTEPH group treated with pulmonary vasodilator therapy before surgery had a significantly lower risk of developing persistent PH than those who did not receive this treatment. We hypothesized that preoperative pulmonary vasodilator treatment could facilitate surgery by making the procedure technically easier for the surgeon and enabling the removal of more fibrotic clots, particularly in patients with lower pulmonary pressures. Our results accounted for potential confounders such as surgical timing (> 5 vs. 5 ≤ years), confirming that surgical technique did not influence outcomes (*p* = 0.85). Interestingly, the number of PE episodes did not affect the risk of persistent PH, suggesting that a history of multiple PEs does not necessarily lead to more distal vasculopathy than a single episode. Moreover, presurgery hemodynamics, such as elevated mPAP or PVR, did not predict the development of persistent PH in either group, which was contrary to what has been reported in the literature (34).

In experienced centers, the perioperative mortality risk following PEA is less than 2.5%, which is attributed to advancements in managing cardiac and pulmonary complications (28). Prior studies have documented the safety of PEA in patients with APS, highlighting a low risk of mortality that is comparable to patients without APS. Rosen et al. (14) reported significant functional and hemodynamic improvements after PEA with a survival rate of approximately 87%. Mortality in their cohort was related to advanced disease and surgical urgency. Long-term follow-up (median: 41 months) revealed that 38% of survivors required pulmonary vasodilators for persistent PH (14). Taş et al. (15) reported a 6.25% risk of late mortality primarily due to coronavirus disease 2019 pneumonia. They also observed persistent PH in 12.5% of their patients during follow-up (mean: 75 months). Camous et al. (27) reported a 30-day mortality rate of 5.9%, with no significant difference between patients with APS-CTEPH and controls.

Our cohort consisted of a moderately ill group of patients, with a mean mPAP of 50 mmHg and a mean PVR of 12 Wood units. According to the literature, these parameters are associated with an increased risk of mortality (35–37). Despite this, our patients achieved statistically significant hemodynamic improvements in both groups (*p* < 0.0001), with no significant difference in mortality between the APS-CTEPH and non-APS CTEPH groups. Post-PEA deaths were recorded at 3.0 years, 5.9 years, 6.5 years, and 10.0 years after surgery. The observed 5-year survival rate after PEA was 98.3%, with a lower limit for true survival of 88.4%, confirming previous findings in the literature (37–39).

Our study thoroughly addressed the outcomes, specifically persistent PH, between the APS-CTEPH and non-APS CTEPH groups. However, this study had several limitations, such as its retrospective nature and small cohort size. Additionally, we observed that there were missing data especially after converting patient records from charts to electronic formats. Patients missing postoperative data who were treated with PEA outside Saudi Arabia were eliminated from some parts of the analysis. Moreover, detailed surgical specimen classification was not available in this retrospective cohort, limiting our ability to accurately characterize disease distribution. Finally, the variability in the timing of follow-up studies, such as RHC and echocardiography before and after surgery, made it difficult to establish a unified timeline for assessing specific outcomes.

## Conclusion

Patients in the APS-CTEPH experienced a greater incidence of immediate postoperative complications and consequently hospitalizations within the 1st year following treatment with PEA. However, the risk of persistent PH or mortality did not increase compared with the patients in the non-APS CTEPH group. Further research involving larger patient cohorts is necessary to validate these findings and to develop strategies for improving outcomes for these patients.

## Data Availability

The original contributions presented in the study are included in the article/Supplementary material, further inquiries can be directed to the corresponding author.
