# Supplementary material for: Pulmonary endarterectomy in antiphospholipid syndrome: a retrospective analysis from the Saudi pulmonary hypertension registry
Source: Front Med (Lausanne). 2026 Jan 14;13:1736115. doi: 10.3389/fmed.2026.1736115 (PMC12848920; doi:10.3389/fmed.2026.1736115)
Supplement: Supplementary file 1 [file Table_1.docx]

**Supplemental Table 1.** Characteristics of the patients diagnosed with chronic thromboembolic pulmonary hypertension and antiphospholipid syndrome.

| Baseline characteristics | APS-CTEPH, *n = 37* |
| --- | --- |
| Type of APS |  |
| Primary | 27 (73.00) |
| Secondary (SLE) | 10 (27.00) |
| Number of positive APS antibodies |  |
| Single | 5 (13.51) |
| Double | 16 (43.24) |
| Triple | 16 (43.24) |
| Immunosuppression |  |
| On therapy | 24 (65.00) |
| HCQ | 21 (87.50) |
| Steroids | 11 (45.80) |
| CellCept | 2 (8.30) |
| Cyclophosphamide | 1 (4.20) |
| Rituximab | 1 (4.20) |

APS: Antiphospholipid syndrome; CTEPH: Chronic thromboembolic pulmonary hypertension; HCQ: Hydroxychloroquine; SLE: Systemic lupus erythematosus.

**Supplemental Table 2.** The detailed antibody distribution of the patients diagnosed with chronic thromboembolic pulmonary hypertension and antiphospholipid syndrome.

| Positive antibody profile | Primary APS, *n =27* | Secondary APS, *n = 10* |
| --- | --- | --- |
| aCL IgG & LA& anti-β2GP IgG | 7 (26) | 6 (60) |
| aCL IgM & LA& anti-β2GP IgM | 3 (10) | 0 (0) |
| aCL IgG & anti-β2GP IgG | 8 (30) | 3 (30) |
| aCL IgG & anti-β2GP IgM | 1 (4) | 0 (0) |
| aCL IgM & anti-β2GP IgM | 2 (7) | 0 (0) |
| aCL IgM & LA | 2 (7) | 0 (0) |
| aCL IgG & LA | 0 (0) | 1 (10) |
| aCL IgM only | 1 (4) | 0 (0) |
| LA only | 1 (4) | 0 (0) |
| anti-β2GP IgG only | 1 (4) | 0 (0) |
| anti-β2GP IgM only | 1 (4) | 0 (0) |

aCL: Anticardiolipin; anti-β2GP: Anti beta glycoprotein; LA: Lupus anticoagulant.

**Supplemental Table 3.** Hemodynamic parameters 1 year before and after treatment assessed by right heart catheterization.

| Hemodynamics | Before PEA | | After PEA | |
| --- | --- | --- | --- | --- |
|  | APS, *n* = 15 | Non-APS, *n* = 25 | APS, *n* = 15 | Non-APS, *n* = 25 |
| RA in mmHg | 14.0 ± 5.0 | 12.0 ± 6.3 | 10.0 ±5.4 | 9.0 ± 4.7 |
| mPAP in mmHg | 46.0 ± 12.9 | 51.0 ± 11.0 | 28.0 ± 9.5 | 33.0 ± 13.0 |
| PAWP in mmHg | 14.0 ± 4.7 | 12.0 ± 5.4 | 12.0 ± 3.4 | 14.0 ± 6.0 |
| PVR in Wood units | 11.0 ± 5.6 | 12.0 ± 8.1 | 4.6 ± 2.4 | 4.7 ± 3.1 |
| CO in L/min | 3.30 ± 1.00 | 3.60 ± 1.30 | 3.90 ± 1.10 | 4.00 ± 0.98 |
| CI in L/min/m^2^ | 1.80 ± 0.40 | 2.00 ± 0.60 | 2.10 ± 0.53 | 2.20 ± 0.45 |
| SvO2 as % | 54.0 ± 11.5 | 58.0 ± 10.0 | 65.0 ± 6.1 | 66.0 ± 6.1 |

APS: Antiphospholipid syndrome; CI: Cardiac index; CO: Cardiac output; mPAP: Mean pulmonary artery pressure; PAWP: Pulmonary artery wedge pressure; PEA: Pulmonary endarterectomy; PVR: Pulmonary vascular resistance; RA: Right atrium; SvO2: Mixed venous oxygen saturation.
